# An apple MYB transcription factor, *MdMYB3,* is involved in regulation of anthocyanin biosynthesis and flower development

**DOI:** 10.1186/1471-2229-13-176

**Published:** 2013-11-07

**Authors:** Sornkanok Vimolmangkang, Yuepeng Han, Guochao Wei, Schuyler S Korban

**Affiliations:** 1 Department of Natural Resources and Environmental Sciences, University of Illinois, 1201 W. Gregory, Urbana, IL 61801, USA; 2 Department of Pharmacognosy, Faculty of Pharmaceutical Sciences, Chulalongkorn University, Bangkok 10330, Thailand; 3 Key Laboratory of Plant Germplasm Enhancement and Specialty Agriculture, Wuhan Botanical Garden, Chinese Academy of Sciences, Moshan, Wuhan 430074, P. R C; 4 Department of Biology, University of Massachusetts, Boston, Boston, MA 02125, USA

**Keywords:** Anthocyanin, Apple, MYB transcription factors, *Malus* × *domestica*, Flavonoids, Reproductive tissues

## Abstract

**Background:**

Red coloration of fruit is an important trait in apple, and it is mainly attributed to the accumulation of anthocyanins, a class of plant flavonoid metabolites. Anthocyanin biosynthesis is genetically determined by structural and regulatory genes. Plant tissue pigmentation patterns are mainly controlled by expression profiles of regulatory genes. Among these regulatory genes are MYB transcription factors (TFs), wherein the class of two-repeats (R2R3) is deemed the largest, and these are associated with the anthocyanin biosynthesis pathway. Although three *MdMYB* genes, almost identical in nucleotide sequences, have been identified in apple, it is likely that there are other R2R3 MYB TFs that are present in the apple genome that are also involved in the regulation of coloration of red color pigmentation of the skin of apple fruits.

**Results:**

In this study, a novel *R2R3 MYB* gene has been isolated and characterized in apple. This *MYB* gene is closely related to the *Arabidopsis thaliana AtMYB3*, and has been designated as *MdMYB3*. This TF belongs to the subgroup 4 R2R3 family of plant MYB transcription factors. This apple *MdMYB3* gene is mapped onto linkage group 15 of the integrated apple genetic map. Transcripts of *MdMYB3* are detected in all analyzed tissues including leaves, flowers, and fruits. However, transcripts of MdMYB3 are higher in excocarp of red-skinned apple cultivars than that in yellowish-green skinned apple cultivars. When this gene is ectopically expressed in *Nicotiana tabacum* cv. Petite Havana SR1, flowers of transgenic tobacco lines carrying *MdMYB3* have exhibited increased pigmentation and accumulate higher levels of anthocyanins and flavonols than wild-type flowers. Overexpression of *MdMYB3* has resulted in transcriptional activation of several flavonoid pathway genes, including *CHS*, *CHI*, *UFGT*, and *FLS*. Moreover, peduncles of flowers and styles of pistils of transgenic plants overexpressing MdMYB3 are longer than those of wild-type plants, thus suggesting that this TF is involved in regulation of flower development.

**Conclusions:**

This study has identified a novel MYB transcription factor in the apple genome. This TF, designated as *MdMYB3*, is involved in transcriptional activation of several flavonoid pathway genes. Moreover, this TF not only regulates the accumulation of anthocyanin in the skin of apple fruits, but it is also involved in the regulation of flower development, particularly that of pistil development.

## Background

Skin color is an important determinant of apple fruit quality. Generally, consumers prefer red-skinned apples as they are perceived to be associated with better taste and flavor [1]. Coloration of apple fruit is attributed to accumulation of anthocyanins, a class of plant flavonoid metabolites. Flavonoids are ubiquitous in plants, and play important roles throughout plant growth, including UV protection, disease resistance, herbivore defense, and providing flowers and seeds with pigmentation to attract pollinators and seed dispersers [2,3]. More importantly, there is increasing evidence that flavonoids benefit human health such as lowering the incidence of cardiovascular disease, obesity, diabetes, pulmonary disease, and cancer [4-8].

The biosynthetic pathway of anthocyanins has been well established, and anthocyanin pathway genes have been isolated and characterized in a variety of model plants such as petunia, snapdragon, and *Arabidopsis *[9,10]. Anthocyanin biosynthesis is genetically determined by structural and regulatory genes. The structural genes are regulated at the transcriptional level by regulatory genes, and thus plant pigmentation patterns are mainly controlled by the expression profiles of regulatory genes [11,12].

Three transcription factors (TFs), including the basic helix-loop-helix (bHLH), R2R3 MYB, and WD40 proteins, predominantly regulate genes in the anthocyanin biosynthesis pathway across all plant species reported to date, including apple [13,14]. MYB TFs have been reported to play diverse functions in controlling pathways such as secondary metabolism, development, signal transduction, and disease resistance in plants [15]. They are classified by the numbers of highly conserved imperfect repeats in the DNA-binding domain, and consisting of either single or multiple repeats. Among these MYB TFs, the class of two-repeats (R2R3) is deemed the largest, with 339 TFs reported in *Arabidopsis *[16], and it is associated with the anthocyanin biosynthesis pathway.

Regulation of R2R3 MYB TFs can occur at different steps of the anthocyanin biosynthesis pathway. For example, R2R3 MYB TFs in perilla (*Perilla frutescens*) control transcription of all structural genes involved in anthocyanin biosynthesis [17]. *MYBA* in grape (*Vitis vinifera*) specifically regulates genes down-stream of anthocyanin production, but not those of earlier steps [18]. As with other transcription factors, regulation of R2R3 MYB TFs could serve to either activate or repress expression of these genes. For example, MYB TFs such as *Arabidopsis PAP1*, *AtPAP2*, *AtMYB113*, and *AtMYB114 *[19,20], grape *VvMYB1a *[21], and *Gerbera* hybrid *GhMYB10 *[22] positively regulate anthocyanin biosynthesis. Suppression of flavonoid accumulation has been observed in transgenic plants overexpressing strawberry *FaMYB1 *[23], *Antirrhinum AmMYB308 *[24], *Arabidopsis AtMYB4 *[25], and *Arabidopsis AtMYBL2* which encode a single-repeat R3-MYB protein [26,27].

In recent years, several studies have been reported on the characterization of structural and regulatory genes involved in fruit coloration in apple (*Malus × domestica* Borkh.). For example, induction of most structural genes in the anthocyanin biosynthesis pathway can significantly increase accumulation of anthocyanin in apple skin [28]. Three transcription factors, *MdMYB10*, *MdMYB1*, and *MdMYBA*, have been isolated and characterized in apple [29-31]. Of the three TFs, *MdMYB10* is responsible for red flesh coloration, while *MdMYB1* and *MdMYBA* control red skin coloration of apple fruit. The three *MdMYB* genes are almost identical in nucleotide sequences, and have been subsequently reported to be of different alleles on linkage group 9 [32,33]. Recently, Chagné et al. [34] have reported that the red-flesh cortex phenotype of apple fruit is associated with enhanced expression of *MYB110a*, a paralog of *MYB10*, and functional analysis of *MYB110a* in tobacco has revealed that it is involved in up-regulation of anthocyanin biosynthesis. Apple fruits vary considerably in color, ranging from yellow, green, or red, along with varied differences in red color pigmentation patterns. It seems unlikely that apple fruit skin red coloration is simply controlled by a single locus.

Isolation and characterization of MYB TFs associated with anthocyanin biosynthesis is an important key step towards understanding and manipulating fruit coloration. In this study, a MYB TF, designated *MdMYB3,* has been identified using an apple expressed sequence tag (EST) database [35] and a BAC-based physical map of the apple genome [36]. The *MdMYB3* gene shows higher levels of expression in exocarp of red-skinned apple cultivars than that of yellowish-green skinned apple cultivars. Transgenic flowers overexpressing *MdMYB3* accumulate higher levels of anthocyanin and have longer peduncles and styles when compared with those of wild-type flowers. These results strongly suggest that *MdMYB3* not only regulates anthocyanin biosynthesis, but is also involved in flower and pistil development.

## Results

### Sequence characterization of *MdMYB3* in apple

**Figure 1:**
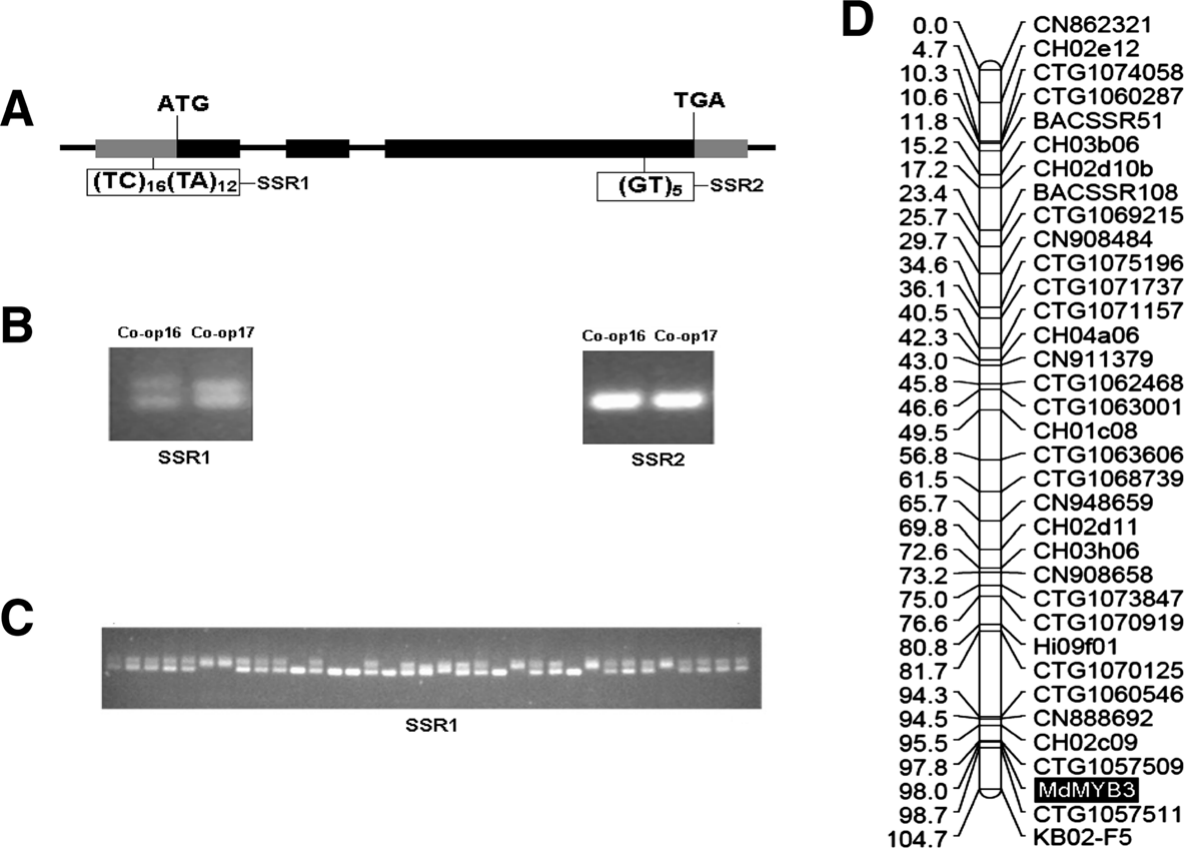
**Sequence characterization and genetic mapping of the *MdMYB3 *gene in apple. A)** Genomic structure of *MdMYB3* gene in apple. The black and gray boxes indicate exons and un-translated region (UTR). SSR1 and SSR2 represent simple sequence repeats, and are present in 5′-UTR and the last exon, respectively. **B)** The identification of polymorphism of SSR1 and SSR2 loci between Co-op 16 and Co-op 17. The SSR1 locus has been successfully used to develop gene-tagged marker for *MdMYB3* in apple. **C)** Segregation of SSR1 locus among F_1_ progenies derived from a cross between 'Co-op 16′ x ′Co-op 17′. The F_1_ population consists of 142 individuals and only partial individuals are presented. **D)***MdMYB3* gene, highlighted in shaded area, is anchored onto linkage group 15 based on the segregation of SSR1 locus among F1 population of 'Co-op 16′ x 'Co-op 17′ cross.

A genomic DNA sequence encoding R2R3 MYB has been isolated from cv. GoldRush. When this sequence is BLASTed against the *Arabidopsis* genome sequence database (http://www.arabidopsis.org/Blast/index.jsp), a best hit to the *AtMYB3* gene is found, and thus the gene is designated as *MdMYB3*. The *MdMYB3* gene consists of three exons and two introns along with two tandem repeats, (TC)_16_(TA)_12,_ designated as SSR1 in the 5′ un-translated region (UTR), as well as a dinucleotide (GT)_5,_ designated as SSR2 in the last exon (Figure 1A). The full-length cDNA of *MdMYB3* is 1,193 bp in size and encodes a putative protein of 310 amino acids along with an ATG start codon, at position 162 of the nucleotide sequence, and a TGA stop codon, at position 1094.

**Figure 2:**
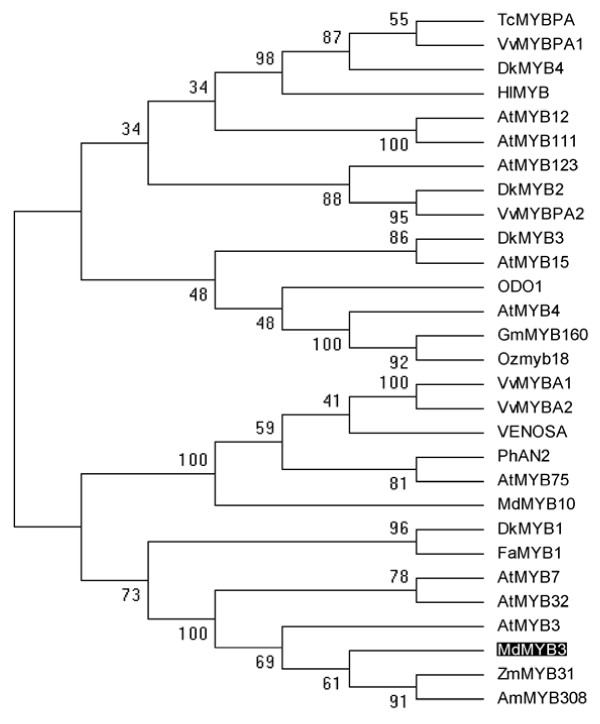
**Phylogenetic tree derived from amino acid sequences of genes encoding R2R3 MYB in plants.** The apple MdMYB3 is shaded. The phylogenetic analysis is performed using the Maximum Parsimony method. Numbers on branches correspond to bootstrap estimates for 1000 replicate analyses with stepwise addition of taxa. The GenBank accession numbers are as follows: *Vitis vinifera VvMYBPA1* (BAD18977), *Vitis vinifera VvMYBPA2* (BAD18978), *Theobroma cacao TcMYBPA* (ADD51352), *Fragaria ananasa* FaMYB1 (AF401220), *Diospyros kaki DkMYB4* (AB503701), *Humulus lupulus HiMYB* (CBI83257), *Arabidopsis thaliana AtMYB123* (Q9FJA2), *DkMYB2* (AB503699), *Arabidopsis thaliana* AtMYB12 (NP_182268), *Arabidopsis thaliana* AtMYB111 (NP_199744), *Diospyros kaki DkMYB3* (AB503670), *Arabidopsis thaliana AtMYB15* (Y14207), *Diospyros kaki DkMYB1* (AB503698), *Petunia x hybrida* ODO1 (AAV98200), *Arabidopsis thaliana AtMYB4* (BAA21619), *Glycine max* GmMYB160 (ABH02907), *Oryza sativa* Ozmyb18 (CAD44612), *Malus × domestica* MdMYB8 (DQ267899), *Arabidopsis thaliana* AtMYB75 (NP_176057), *Malus × domestica* MdMYB10 (ABB84753), *Petunia hybrida* PhAN2 (AAF66727), *Zea mays* ZmMYB31 (NM_001112479), *Antirrhinum majus* VENOSA (ABB83828), *Vitis vinifera* VvMYBA1 (BAD18977), and *Vitis vinifera* VvMYBA2 (BAD18978).

**Figure 3:**
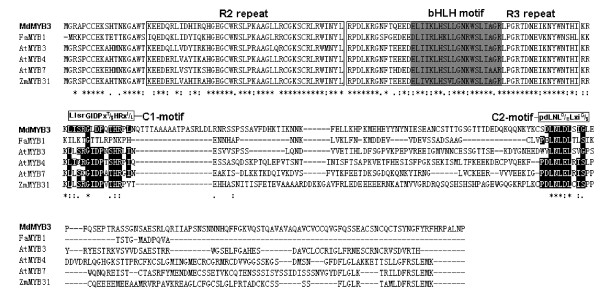
**Comparisons of deduced amino acid sequences of MdMYB3 in apple and R2R3 MYB proteins from other plants using ClustalW2 program (**http://www.ebi.ac.uk/Tools/msa/clustalw2/**).** The apple MdMYB3 is highlighted in bold. R2 and R3 repeats are boxed. The bHLH motif is indicated in gray color, while C1 and C2 motifs are highlighted in black color. Conserved sequences with 100%, 80%, and 60% identity are marked with asterisks, two dots, and one dot, respectively.

Phylogenetic analysis based on amino acid sequences of R2R3 MYB encoding genes from different plants indicates that *MdMYB3* is very closely related to *Arabidopsis AtMYB3*, *AtMYB4*, and *AtMYB7* (Figure 2), belonging to the subgroup 4 R2R3 family of plant MYB transcription factors [37]. Amino acid sequence alignment between MdMYB3 and several previously reported MYB transcription factors, including *Arabidopsis* AtMYB3, AtMYB4, AtMYB7, and AtMYB32, *Fragaria ananasa* FaMYB1, and *Zea mays* ZmMYB31 have revealed that MdMYB3 consists of both R2 and R3 DNA-binding domains (Figure 3). An R/B-like bHLH binding motif ([D/E]Lx_2_[R/K]x_3_Lx_6_Lx_3_R, previously reported by Zimmermann et al. [38], is identified in the R3-DNA binding domain of MdMYB3 (Figure 3). Moreover, MdMYB3 contains two conserved motifs LIsrGIDPx^T^/_S_HRx^I^/_L_ (C1-motif) and pdLNL^D^/_E_Lxi^G^/_S_ (C2-motif) at the C-terminus, previously found in R2R3 MYB encoding genes of subgroup 4. However, the C-terminal downstream of the two conserved motifs shows high divergence. MdMYB3 has a 50% amino acid sequence identity with AtMYB3.

### Mapping of the *MdMYB3* gene onto the apple genetic map

Two pairs of primers flanking the SSR1 and SSR2 repeats within the *MdMYB3* gene were designed and used to screen the two parents of the F_1_ population of the ′Co-op 16′ x 'Co-op 17′ cross. The two parents were found to be heterozygous and homozygous at SSR1 and SSR2 loci, respectively (Figure 1B). The primers flanking the SSR1 locus was then selected to screen F_1_ progenies of 'Co-op 16′ x 'Co-op 17′ cross. As a result, three genotypes, designated 'hh’ (upper band), 'hk’ (upper and lower bands), and 'kk’ (lower band), respectively, were identified for the SSR1 locus among this progeny (Figure 1C). Based on our recently constructed apple genetic linkage map [36], the apple *MdMYB3* gene was anchored onto linkage group 15 (Figure 1D).

### Expression profiles of *MdMYB3* in apple

**Figure 4:**
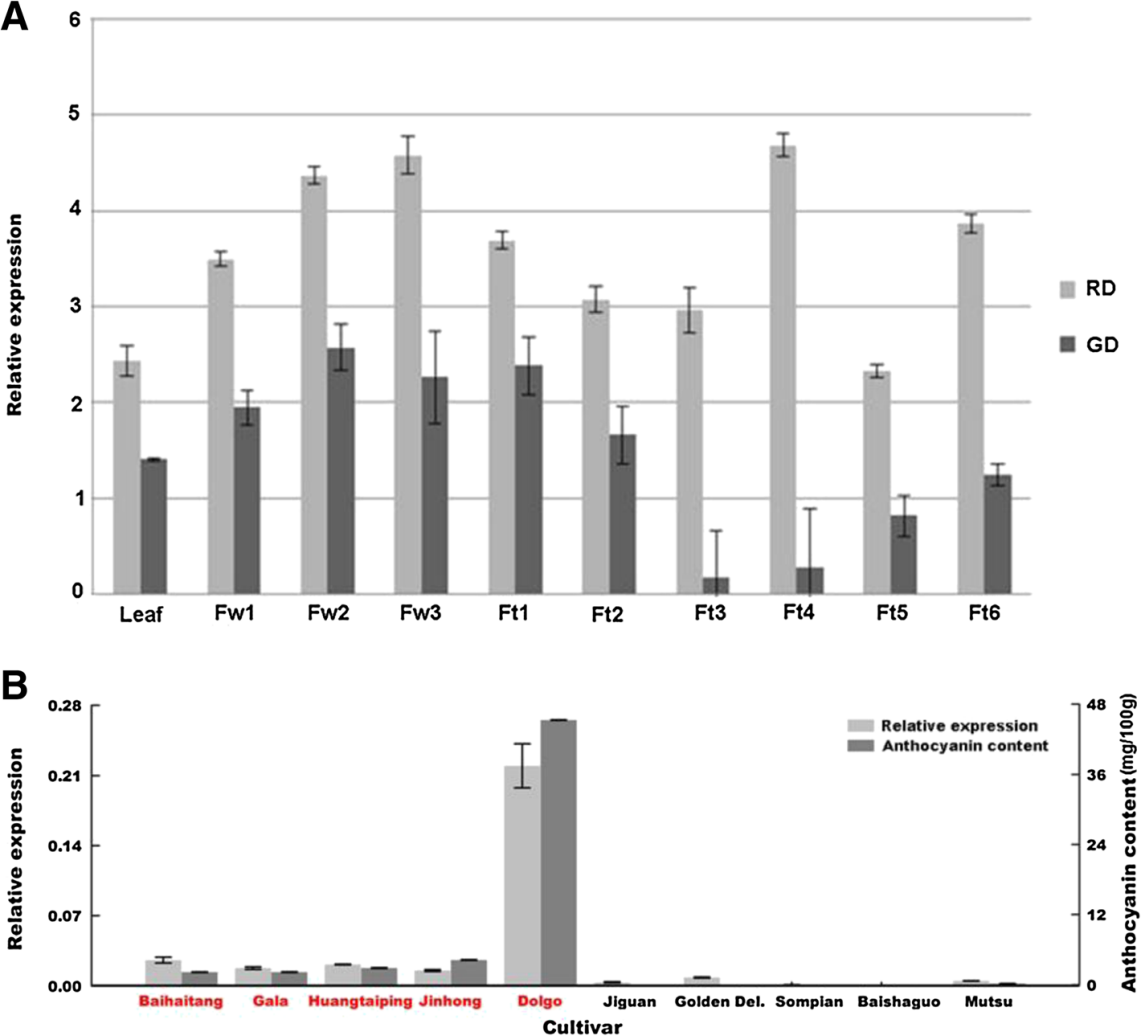
**Expression profiles and anthocyanin content of *MdMYB3 *in various tissues of apple cultivars using qRT-PCR. A)** Expression of *MdMYB3* in Red Delicious and Golden Delicious apple cultivars. Abbreviations are listed as followings: Fw1: Flower buds at the pink stage; Fw2: flower buds at the balloon stage; Fw3: flowers at full bloom; Ft1: 9 DAP; Ft2: 16 DAP; Ft3: 44 DAP; Ft4: 104 DAP; Ft5: 145 DAP; Ft6: 166 DAP; RD: 'Red Delicious’; GD: 'Golden Delicious’. **B)** Expression of *MdMYB3* and anthocyanin contents in red-skinned cultivars (written in red color font) and yellowish-green skinned cultivars (in black color font). Normalization was made to expression of the actin gene, and values are means of three technical replicates.

Expression profiles of *MdMYB3* in apple cvs. Red Delicious (red-skinned fruit) and Golden Delicious (yellow-skinned fruit) were investigated. Quantitative real-time (qRT)-PCR analysis revealed that *MdMYB3* transcripts accumulated in all analyzed tissues, including leaves, flowers, and fruits (Figure 4A). Overall, transcript levels of *MdMYB3* in all analyzed tissues were higher in cv. Red Delicious than those in cv. Golden Delicious. Accumulation of *MdMYB3* transcripts in flowers of 'Red Delicious’ increased throughout flower development and reached a peak at full-bloom (completely open flowers), while transcripts of *MdMYB3* in flowers of 'Golden Delicious’ showed a peak at the balloon stage (closed, yet ballooned flower buds), and then slightly decreased until full-bloom (fully-open flowers). Transcripts of *MdMYB3* in fruits of both cvs. Red Delicious and Golden Delicious increased during early stages of development, but then decreased slightly at 44 days after pollination (DAP). Subsequently, transcript accumulation of *MdMYB3* in fruits of cv. Golden Delicious gradually increased until maturity; whereas, those of cv. Red Delicious peaked at fruit stage IV, and remained relatively high at fruit maturity.

Subsequently, a total of 10 apple cultivars were selected and used to investigate the association of *MdMYB3* gene expression with anthocyanin accumulation in excocarp of fruits at maturity. Overall, *MdMYB3* transcripts were highly expressed in excocarp of red-skinned fruits, but were either low or undetectable in yellowish-green skinned fruits (Figure 4B). These expression profiles were accompanied with similar anthocyanin content profiles in cortex tissues of these apple cultivars (Figure 4B). This finding further confirmed that *MdMYB3* was involved in anthocyanin accumulation in apple.

### Functional analysis of *MdMYB3* in tobacco

**Figure 5:**
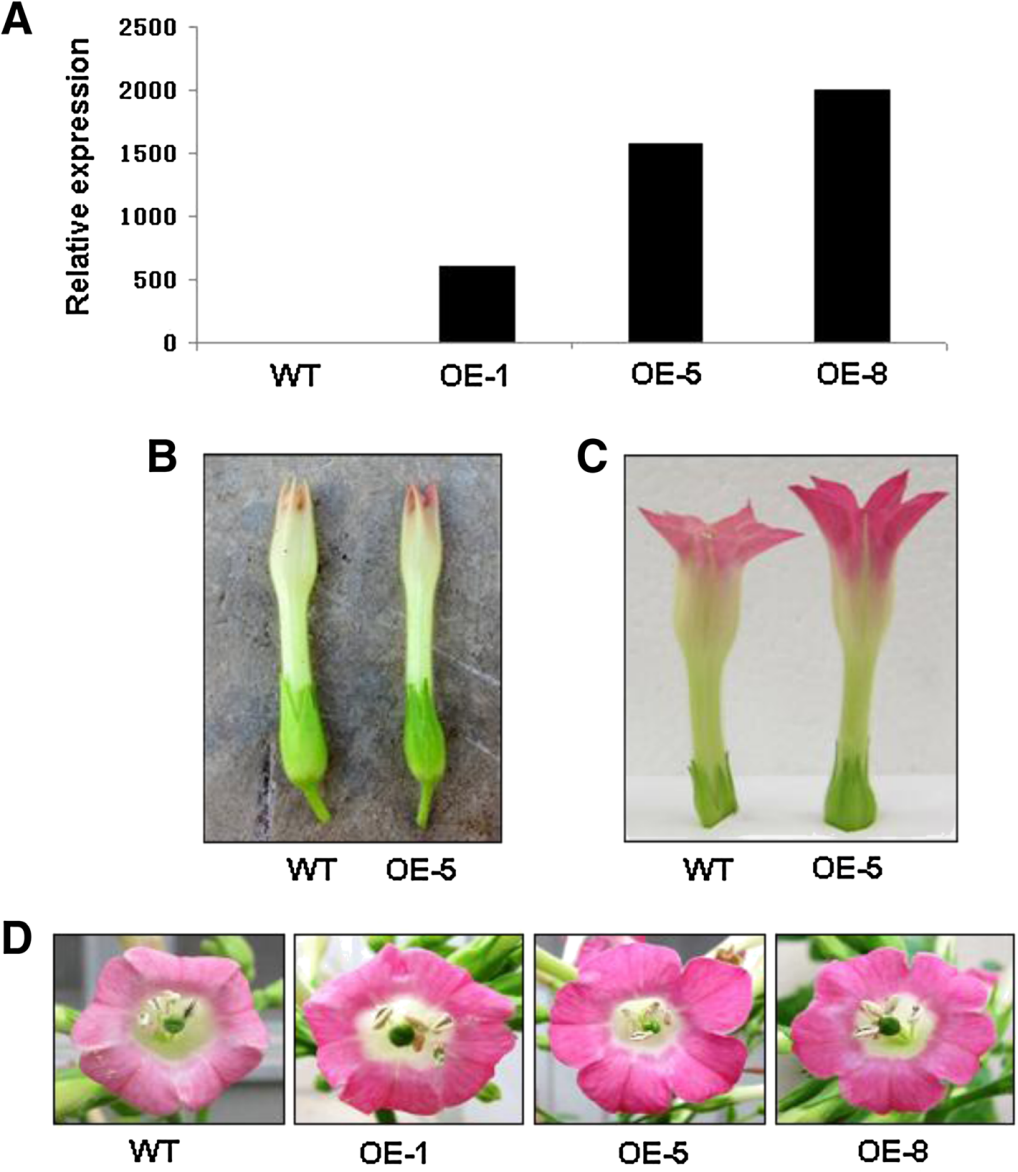
**Tobacco flowers of wild-type and three transgenic lines, OE-1, OE-5, and OE-8, carrying *MdMYB3*. A**. Transcript levels of *MdMYB3* in flowers of transgenic lines and wide type (WT) control plants. **B**. Flowers of wild-type (left) and transgenic line MdMYB3-5 (right) during early flowering stage; **C**. Flowers of wild-type (left) and transgenic line MdMYB3-5 (right) at early bloom; **D**. Flowers of WT and transgenic lines at full bloom.

The coding sequence of *MdMYB3*, driven by the constitutive promoter of cauliflower mosaic virus (CaMV) 35S, was introduced into tobacco, and three T_2_ transgenic lines, designated as OE-1, OE-5, and OE-8, were generated. Flowers of transgenic lines showed darker color pigmentation than those of wild-type plants. For example, corolla of flowers of plants of line OE-5 began to show pink coloration during earlier stages of flower development than those of wild-type plants (Figure 5B). Subsequently at early bloom, corolla of flowers of OE-5 were almost dark pink while those of wild-type were light pink (Figure 5C). Corolla of flowers of all three transgenic lines continued to show increased pigmentation until full-bloom (completely open flowers), and showed markedly darker pink coloration, almost fuchsia, than those of wild-type plants (Figure 5D).

LC/MS/MS analysis revealed that transgenic flowers of tobacco contained higher levels of flavonoids than wild-type flowers (Table 1). For example, levels of cyanidin in transgenic flowers were 2- to 4-fold higher than those of wild-type flowers. Moreover, levels of two proanthocyanidin components, catechin and epicatechin, in transgenic flowers were 1.1- to 1.4-fold and 1.3- to 4.5-fold, respectively, higher than those of wild-type flowers. These findings suggested that *MdMYB3* was involved in the regulation of flavonoid biosynthesis in tobacco flowers.

**Table 1:** Analysis of flavonoid contents in tobacco flowers of wild-type and transgenic lines using LC/MS/MS method*

**Flowers**	**Flavonol (ng/g)**		**Anthocyanidin (ng/g)**	**Proanthocyanidin (ng/g)**	
	**Kaempferol**	**Quercetin**	**Cyanidin**	**Catechin**	**Epicatechin**
WT	60.57	71.80	1722	4.80	6.47
MdMYB3-1	53.93	88.83	3060	5.60	8.60
MdMYB3-5	73.50	136.67	7707	6.20	29.13
MdMYB3-8	79.27	114.67	4513	6.93	18.93

*Kaempferol, quercetin, cyanidin, catechin, and epicatechin were used as standards. All data correspond to mean values of three biological replicates.

**Figure 6:**
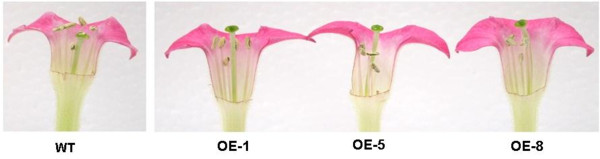
**Tobacco flowers of wild-type and transgenic lines carrying *MdMYB3*.** In transgenic lines, styles are elongated leading to stigmas that protrude above anthers; whereas, styles in pistils of wild-type tobacco flowers are shorter, and stigmas are positioned below anthers.

In addition to flower color pigmentation, differences in other morphological traits, including lengths of flowers and lengths of styles of pistils, were also observed between wild-type and transgenic lines. For example, at full bloom, flowers of tobacco plants of transgenic line OE-5 were longer, on average 8-10 mm longer, than those of wild-type plants (Figure 5C). Moreover, lengths of styles of pistils of transgenic flowers were also longer, on average 10- 14 mm longer, than those of wild-type flowers, thus positioning stigmas above anthers (Figure 6).

### Expression profiles of structural genes of phenylpropanoid and flavonoid pathways in tobacco transgenic flowers overexpressing *MdMYB3*

**Figure 7:**
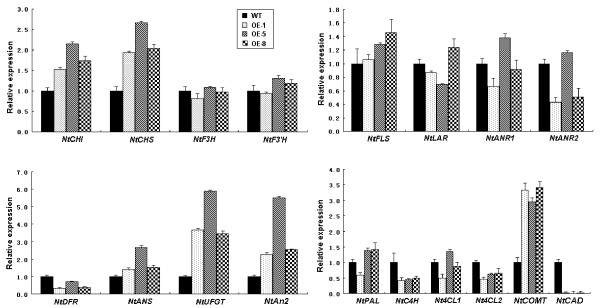
**Expression analysis of phenylpropanoid and flavonoid pathway genes in flowers of T**_**2 **_**transgenic tobacco lines carrying *MdMYB3 *using qRT-PCR.** Normalization was adjusted to expression of an apple *actin* gene, and values are means of three technical replicates. Transcript levels in flowers of three transgenic lines were quantified relative to those present in flowers of wild-type plants. Abbreviations correspond to the following: CHI: chalcone isomerase; CHS: chalcone synthase; F3H: flavonoid 3-hydroxylase; F3′H: flavonoid 3′-hydroxylase; FLS: flavonol synthase; DFR: dihydroflavonol reductase; LAR: leucoanthocyanidin reductase; ANS: anthocyanidin synthase; UFGT: glucose transferase; ANR: anthocyanidin reductase; C4H: cinnamate-4-hydroxylase; 4CL: 4-coumaroyl-CoA ligase; PAL: phenylalanine ammonia lyase; COMT: caffeoyl-CoA o-methyltransferase; and CAD: cinnamyl alcohol dehydrogenase.

Transcripts of 15 structural genes involved in biosynthesis pathways of both phenylpropanoid and flavonoid were evaluated in flowers of both wild-type and transgenic tobacco plants (Figure 7). Of these 15 genes, flavonoid-specific genes, including *NtCHI*, *NtCHS*, NtANS, *NtUFGT*, *NtAn2*, and *NtCOMT* showed similar patterns in transcript accumulation for all three transgenic lines as they were significantly up-regulated compared to those of wild-type flowers (Figure 7). Moreover, transcripts of *NtDFR* and three phenylpropanoid pathway genes including *NtC4H*, *Nt4CL2*, and *NtCAD* exhibited similar patterns of gene expression as they were all down-regulated in flowers of all three transgenic lines compared to those of wild-type plants. All remaining genes showed different patterns of gene expression in flowers transgenic lines when compared to those wild-type plants.

## Discussion

Multiple R2R3 TFs have been reported to be involved in anthocyanin biosynthesis in many plant species [14]. However, it is not clear whether or not there are additional loci, other than the *MdMYB10* locus. Using second-generation resequencing, Chagné et al. [34] have identified 33 SNPs within a 60-kb region surrounding the two alleles *MYB110a* and *MYB110b* that are involved in the regulation of red flesh (or cortex) color pigmentation of apple fruit. Chromosomal location of *MYB110a* has been attributed to whole-genome duplication event that occurred during the evolution of apple within the Maloideae family [34]. This is to be expected as integration of an apple physical and genetic maps have demonstrated presence of both genome-wide and segmental duplications in the apple genome and providing further insights into the complex polyploid ancestral origin of the apple [36]. In this study, a novel R2R3 MYB transcription factor *MdMYB3* in apple has been isolated, and its ectopic expression in tobacco flowers indicates that it is involved in flower coloration, peduncle length, and style/stigma development.

### The apple *MdMYB3* is involved in regulation of the anthocyanin biosynthesis pathway

When the coding DNA sequence of *MdMYB3* was BLASTed against the *Arabidopsis* genome sequence database (http://www.arabidopsis.org/Blast/index.jsp), hits to subgroup 4 of MYB TFs were detected, including those of *AtMYB3*, *AtMYB7*, and *AtMYB4* with E-values of 3e-25, 2e-23, and 1e-21, respectively. Although *AtMYB3* and *AtMYB7* have not been functionally characterized, *AtMYB4* has been reported to function as a repressor of the lignin biosynthesis pathway [25]. It is noteworthy to point out that the *MdMYB3* gene identified in this study is different from MYB repressors reported by Lin-Wang et al. [39]. Although, it is originally anticipated that *MdMYB3* would have a similar function to that of *AtMYB4*, flowers of transgenic tobacco lines overexpressing *MdMYB3* have accumulated higher levels of anthocyanin than those of wild-type plants, resulting in increased color pigmentation. Analysis of gene expression profiles in flowers of T_2_ transgenic lines has further revealed that *MdMYB3* contributes to activation of *NtCHI*, *NtCHS*, *NtANS*, *NtUFGT*, and *NtAn2* genes, and some degree of repression of the *NtDFR* gene. These results clearly indicate that *MdMYB3* is involved in regulation of the anthocyanin biosynthesis pathway. The functionality of subgroup 4 MYB genes involved in activation of anthocyanin synthesis has also been reported in maize [40]. The maize *ZmMYB31* gene is closely related to *MdMYB3*, and its overexpression in *Arabidopsis* has been reported to enhance transcription of anthocyanin pathway genes including those of *CHI*, *F3H*, *F3′H*, and *DFR*.

More recently, an R2R3 MYB regulator from tobacco, *NtAn2*, has been isolated and reported to be a key gene controlling anthocyanin production in reproductive tissues of tobacco [41]. Interestingly in this study, expression levels of *NtAn2* in flowers of transgenic tobacco lines overexpressing *MdMYB3* are significantly higher than those of wild-type plants (Figure 7). Zhao et al. [42] have reported that *Arabidopsis MYB4* contains a MYB binding site motif A(A/C)C(A/T)A(A/C)C. The MYB4 protein can interact with its own MYB binding motif, thus regulating its own expression through an autoregulatory mechanism. We have analyzed the promoter sequence of the tobacco *NtAn2* (Genbank accession no. FJ472650), and found a MYB binding site motif, AACTAAC, located at -817 to -811 bp upstream of the start codon. To clarify the role of *MdMYB3* in regulation of anthocyanin biosynthesis, it is worthy to investigate as whether or not the MdMYB3 protein can bind to the promoter region of the *NtAn2* that may subsequently lead to transcriptional activation of *NtAn2*. However, it cannot be ruled out that *MdMYB3* may positively regulate expression of flavonoid structural genes such as *CHS* and *UFGT*. Previously it has been reported that expression levels of *UFGT* in the red-skinned cv. Red Delicious are significantly higher than those in the yellow-skinned cv. Golden Delicious [43]. Herein, we further demonstrate that *MdMYB3* strongly activates transcription of the *UFGT* gene in flowers of transgenic tobacco lines. Further studies are needed to determine whether or not the MdMYB3 protein can bind to the promoter region of *MdUFGT* and contributing to increased accumulation of anthocyanin.

In this study, ectopic expression of *MdMYB3* in tobacco has contributed to anthocyanin accumulation, predominantly detected in reproductive tissues. Similar findings have been reported for transgenic tobacco lines carrying an apple *MdMYBA* gene [30] as well as transgenic *Arabidopsis* lines carrying an apple *MdMYB1*[29]. In contrast, several other anthocyanin-related TFs such as the grape *VlMybA1-1*, Gerbera *GMYB10*, tomato *LeANT1*, and *Arabidopsis AtPAP1* are capable of inducing anthocyanin accumulation in whole plants [18,19,22,44]. Previously, it has been reported that these *MYB* genes are capable of activating bHLH transcription, and thus forming a complex with bHLH to promote accumulation of anthocyanin [45,46]. In this study, *MdMYB3* may not function as a bHLH activator. Instead, it may combine with a tissue-specific bHLH to activate transcription of either *NtAn2* or other anthocyanin pathway genes, resulting in anthocyanin accumulation in flowers.

In apple, *MdMYB10* and *MdMYB1* strongly up-regulate most flavonoid structural genes, including *MdCHI*, *MdCHS*, *MdF3H*, *MdLDOX*, *MdDFR*, and *MdUFGT *[29,31]. In contrast in this study, *MdMYB3* activates transcription of *NtCHI*, *NtCHS*, and *NtUFGT* genes in tobacco flowers, but it inhibits transcription of the *NtDFR* gene. Moreover, expression levels of structural genes such as *NtF3H*, *NtF3′H*, *NtFLS*, and *NtLDOX* in flowers of transgenic tobacco in this study are on average similar to those detected in flowers of wild-type plants. These findings suggest that the role of *MdMYB3* in regulation of the anthocyanin biosynthesis pathway may be different from those of *MdMYB10* and *MdMYB1*. This is consistent with the phylogenetic analysis that revealed that *MdMYB3* is separated from *MdMYB10*/*MdMYB1*. Moreover, *MdMYB3* is located on chromosome 15, and is expressed at higher levels in the cortex of red-skinned apple cultivars than in yellowish-green skinned apple cultivars. Therefore, it seems that MYB TFs other than *MdMYB10*/*MdMYB1*, located on chromosome 9, may also be involved in the regulation of apple red skin coloration.

The phylogenetic analysis indicates that *MdMYB3* has a close relationship with *Arabidopsis AtMYB12* and *AtMYB111.* These two *Arabidopsis* MYB TFs positively regulate expression of *CHS*, *FLS*, and *F3H* genes, and are thus responsible for accumulation of flavonol [47]. Similarly in this study, *MdMYB3* is activating transcription of *NtCHS* and *NtFLS* in flowers of transgenic tobacco. Overall, levels of kaempferol and quercetin in flowers of transgenic tobacco plants are higher than those in wild-type plants (Table 1). Therefore, it seems that the apple *MdMYB3* gene is also involved in positive regulation of flavonol accumulation. Interestingly, the *Arabidopsis AtMYB12* does not contain an R/B-like bHLH binding motif, thus it functions independently of a bHLH cofactor [48]. However, MdMYB3 has an R/B-like bHLH binding motif, suggesting it can form a complex with bHLH to regulate anthocyanin biosynthesis. Thus, it is likely that the apple *MdMYB3* gene has functionally diverged from the *Arabidopsis AtMYB12*.

### Functional divergence of the apple *MdMYB3* from its closely related genes

The *MdMYB3* gene is clustered together with the strawberry *FaMYB1* (Figure 2). Ectopic expression of *FaMYB1* in tobacco inhibits accumulation of both anthocyanins and flavonols, resulting in strong reduction in flower pigmentation [23]. Overexpression of *FaMYB1* in tobacco flowers down-regulates transcription of late flavonoid biosynthesis genes. In contrast, the *MdMYB3* gene in this study strongly up-regulates expression of genes involved in anthocyanin and flavonol synthesis, including *CHS*, *CHI*, *UFGT*, *ANS*, and *FLS* genes. FaMYB1 contains a conserved C2-motif pdLNL^D^/_E_Lxi^G^/_S_ at the C-terminal, which is responsible for repression of transcription [25]. The C2-motif is also present in the C-terminus of MdMYB3, but its first amino acid sequence has been changed from Phe to Ser (Figure 3). It remains unclear if a single amino acid substitution is responsible for observed functional differences between MdMYB3 and FaMYB1.

The apple *MdMYB3* gene is also closely related to the maize *ZmMYB31* gene and the *Arabidopsis AtMYB32* and *AtMYB4* genes*.* These three latter genes have been previously reported to function as repressors in the lignin biosynthesis pathway. For example, *AtMYB4* and *AtMYB32* down-regulate expression of *C4H* and *COMT* genes, respectively, and are thus deemed as repressors of lignin biosynthesis [25,49]. It is likely that *ZmMYB31* inhibits transcription of *COMT*, *F5H*, *C3H*, and *4CL* genes, resulting in reduced levels of lignin biosynthesis [40]. Thus, it has been presumed that *MdMYB3* will have a similar function to those of its closely related genes. As expected, overexpression of *MdMYB3* in tobacco flowers significantly represses transcription of genes involved in the lignin biosynthesis pathway such as *C4H* and *4CL2*; moreover, it also severely inhibits expression of the *CAD* gene involved in monolignol biosynthesis (Figure 7). However, expression of *NtCOMT* in flowers of transgenic lines overexpressing *MdMYB3* is significantly higher than that in wild-type plants (Figure 7). These results suggest that *MdMYB3* may have functionally diverged from subgroup 4 MYB genes such as those of *AtMYB4* and *AtMYB32*.

### The apple *MdMYB3* is involved in regulation of style development in pistils of flowers

It has been reported that changes in expression levels of *AtMYB32* and *AtMYB4* can influence pollen development by changing the flux of the phenylpropanoid pathway, and influencing composition of pollen wall [49]. For example, an *AtMYB32* insertion mutant of *Arabidopsis* shows abnormal pollen grains that are either partially or completely devoid of cellular contents [49]. In this study, the subgroup 4 R2R3 family is also involved in the development of reproductive tissues. Transgenic tobacco lines expressing *MdMYB3* have developed flowers with longer peduncles and longer styles than those of wild-type flowers. Of particular interest are those elongated styles as they result in stigmas that are positioned anthers of a flower. This suggests that *MdMYB3* is also involved in the regulation of pistil development. Similar observations have been previously reported for an *Antirrhinum AmMYB308*, a homolog of *MdMYB3 *[24]. When *AmMYB308* is overexpressed in transgenic tobacco, elongated styles have been observed, resulting in protruded stigmas and contributing to infrequent self-pollination [24]. However, unlike morphological observations noted in this study, flowers of transgenic tobacco overexpressing *AmMYB308* are smaller in size and accumulate lower levels of anthocyanin than those of wild-type tobacco.

Taken together, this study demonstrates that *MdMYB3* plays important and multiple roles in plant growth and development. Based on functional analysis of transgenic tobacco lines, overexpression of *MdMYB3* in tobacco flowers enhances accumulation of anthocyanins and increases length of peduncles and more importantly lengths of styles.

## Conclusions

In this study, a new R2R3 MYB transcription factor (TF), *MdMYB3*, involved in the anthocyanin biosynthesis pathway was identified in the apple genome. This TF has been characterized and mapped onto Linkage group (LG) 15 of the apple genetic map. Transcripts of this TF are detected in leaves, flowers, and fruits; however, transcripts of MdMYB3 are higher in excocarp of red-skinned apple cultivars than that in yellowish-green skinned apple cultivars thus regulating accumulation of anthocyanin accumulation in the skin of apple fruit. Ectopic expression of this TF in tobacco revealed that it has a regulatory role by activating the transcription of NtAn2 and thus inducing several flavonoid biosynthesis pathway genes. More interestingly, this TF is also involved in floral development by modifying pistil length in flowers.

## Methods

### Plant material

Leaves, flowers, and fruits at different stages of development were collected from trees of apple cvs. Red Delicious and Golden Delicious. In addition, fruits of cvs. Baihaitang, Gala, Huangtaiping, Jinhong, Dolgo, Jiguan, Golden Delicious, Sompain, Baishaguo, and Mutsu were also collected at maturity.

### Isolation of genomic DNA encoding MdMYB3 in apple

*Arabidopsis AtMYB3* (AT1G22640) was BLASTed against our apple EST database (http://titan.biotec.uiuc.edu/cgi-bin/ESTWebsite/estima_start?seqSet=apple), and a homologous EST contig (accession no. Apple_0223.1923.C1.Contig3505) was identified. The EST contig sequence was then BLASTed against apple EST database in NCBI, and an EST sequence (GenBank accession no. CO868594) containing both R2 and R3 domains was recovered. Based on the EST sequence, a pair of primers (5′-GGGAGAGCACCTTGTTGTGAG-3′/5′-GATCTCGTTGTCGGTTCTTCC-3′) was then designed and subjected to screen BAC library of cv. GoldRush using a PCR-based screening method as previously described by Xu et al. [50]. The reaction consisted of 94°C for 3 min, followed by 33 cycles of 94°C for 35 s, 55°C for 30 s, 72°C for 60 s, and followed by a final 8 min extension at 72°C. A positive BAC clones was randomly selected and subjected to sequencing to recover genomic sequence encoding MdMYB3 in apple.

### Recovery of cDNA sequence encoding MdMYB3 in apple

The genomic sequences encoding MdMYB3 were analyzed using FGENESH-M program (http://www.softberry.com), and an open reading frame (ORF) was predicted. A pair of primers (5′-GGAGAGCACCTTGTTGTGAG-3′/5′-ACTGACAATTGCTGCATGCC-3′) was designed based on the predicted ORF, and used to amplify cDNA from leaves of cv. GoldRush. The PCR product was sequenced, and a cDNA fragment 872 bp in size was recovered. The cDNA fragment sequence was BLASTed against NCBI EST database (http://blast.ncbi.nlm.nih.gov/Blast.cgi), and a cDNA containing the full coding region was identified. Subsequently, a pair of primers (5′-CTGATCCAGAAGAAGAAACAGATG-3′/5′-TGGATTCAAAGCAGGTCTGTG-3′) was designed to amplify the full coding region of *MdMYB3* from cv. GoldRush to further verify the predicted ORF.

### Expression vector construction and tobacco transformation

A pair of primers (5′-TGAC*TCTAGA*CTGATCCAGAAGAAGAAAC-3′/5′-ATAC*GAGCTC*TGGATTCAA AGCAG-3′) was designed to amplify the coding region of *MdMYB3* using the proofreading DNA polymerase Platinum® *Pfx* (Invitrogen) following the manufacturer′s instructions. Forward and reverse primers contained *Xba*I and *Sac*I restriction sites at the 5′ end, respectively. The blunt-end PCR product was ligated into the pCR®-Blunt vector using Zero Blunt® PCR cloning kit (Invitrogen) according to the manufacturer′s protocol. The expression vector was confirmed by direct sequencing. The coding sequence of *MdMYB3* was introduced into the pBI121 cloning vector, and the construct was used for *Agrobacterium*-mediated transformation of tobacco (*Nicotiana tabacum* cv. Petite Havana SR1) as described by Han et al. [43]. T1 seed from three confirmed independent transgenic T0 lines overexpressing *MdMYB3* and carrying a single copy of the transgene, including OE-1, OE-5, and OE-8 were selfed to generate T2 plants.

Wild-type and T2 transgenic tobacco plants were grown in the greenhouse, and flowers at full-bloom (completely open flowers) were collected for analysis of gene expression as well as for analysis of contents of flavonoid compounds. Upon collection, all samples were frozen in liquid nitrogen and stored at -80°C until needed.

### Mapping of the *MdMYB3* gene onto the apple linkage map

An SSR marker within a 5′ un-translated region of *MdMYB3* was used to screen an F_1_ mapping opulation derived from a cross between 'Co-op 16′ and 'Co-op 17′. The primer sequences of the SSR marker were as follows: forward 5′-TCACCTCTTCAAACAACACACC-3′ and reverse 5′-TGCTCTCCCCATCTGTTTCT-3′. The PCR product was run on 2% (w/v) metaphor gel. The linkage map was constructed using JoinMap version 4.0, according to Han et al. [36].

### Real-time PCR analysis

Total RNA from leaf and flower tissues were extracted using an RNAqueous Kit (Ambion) according to the manufacturer’s instructions. RNA from fruit tissues was isolated according to the protocol described by Gasic et al. [51]. Total RNA (2 μg) from each tissue was treated with *DNase*I (Invitrogen), and used for cDNA synthesis. The first-strand cDNA synthesis was performed with Oligo (dT) primer using the SuperScript III RT kit (Invitrogen), according to the manufacturer’s instructions. Specific primers for *MdMYB3* and each flavonoid-related gene were designed using Biology Workbench version 3.2 (http://workbench.sdsc.edu). Specific primer sequences and accession numbers of genes used to design primers have been listed in Additional file 1: Table S1.

The SYBR Green real-time PCR assay was carried out in a total volume of 25 μl, consisting of 12.5 μL of 2× SYBR Green I Master Mix (Applied Biosystems), 0.2 μM (each) specific primers, and 100 ng of template cDNA. The amplification program consisted of 1 cycle of 95°C for 10 min, followed by 40 cycles of 95°C for 15 s, and 60°C for 1 min. The fluorescent product was detected at the last step of each cycle. Following amplification, melting temperatures of PCR products were analyzed to determine specificity of the PCR product. Melting curves were obtained by slow-heating at 0.5°C/sec, from 60°C to 90°C, while continuously monitoring the fluorescence signal. A negative control without a cDNA template was run with each analysis to evaluate the overall specificity. Amplifications were carried out in a 96-well plate in a 7300 Real Time PCR System (Applied Biosystems). All experimental samples were run in triplicates. An apple *Actin* gene was used as a constitutive control. Differences between the cycle threshold (*Ct*) of the target gene and the *Actin* gene were used to obtain relative transcript levels of the target gene, and calculated as 2 exp-(*Ct*_target_ - *Ct*_actin_).

### Flavonoid analysis

Anthocyanins and flavonols were extracted from 50 mg of finely ground tissue in 1 ml 1% HCl/methanol (v/v), at room temperature in the dark, with continuous shaking for 1 h, and centrifuged at 13,000 rpm for 15 min. An aliquot of 100 μL of the supernatant was transferred to a fresh tube, and acid-hydrolyzed by adding 30 μL of 3 N HCl, and incubated at 70°C for 1 h in a thermal cycler (Thermo Hybaid MBS 0.25 s, Thermo Scientific). Proanthocyanins (PAs) were extracted using 1 ml 70% (v/v) acetone containing 0.1% (w/v) ascorbate, and incubated at room temperature for 24 h in darkness as described by Takos et al. [29]. The extract was centrifuged at 13,000 rpm for 15 min at room temperature, and the clear supernatant was transferred to a new tube. An aliquot of 200 μL extract was dried at 35°C, and resuspended in 100 μL of 1% (v/v) HCl-methanol and 100 μL of 200 mM sodium acetate (pH 7.5).

Flavonoid contents were determined using LC/MS/MS along with use of commercial standards for kaempferol, quercetin, cyanidin, catechin, and epicatechin (Sigma). The LC/MS/MS analysis was performed on a 5500 QTRAP mass spectrometer (AB Sciex) equipped with a 1200 Agilent HPLC Analyst (version 1.5.1, Applied Biosytems) for data acquisition and processing. A Phenomenex column (3 μ C6-Phenly 11A, 4.6 × 50 mm) was used for separation. The HPLC flow rate was set at 0.3 mL/min, and HPLC mobile phases consisted of A (0.1% formic acid in H_2_O) and B (0.1% formic acid in acetonitrile). The autosampler was maintained at 5°C. The gradient for catechin and epicatechin was as follows: 0 min, 90% A; 10 min, 50% A; 13-18 min, 0% A; and 18.1-25 min, 90% A. The injection volume was 20 μl. The gradient for cyanidin, kaempferol, and quercetin was as follows: 0 min, 70% A; 7-12.5 min, 0% A; and 13-20 min, 70% A. The injection volume was 10 μL. The mass spectrometer was operated with positive electrospray ionization. Multiple reaction monitoring (MRM) was used to quantify catechin and epicatechin (m/z 291.0-- > 139.2), cyanidin (m/z 287.2-- > 213.2), kaempferol (m/z 287.1-- > 153.2), and quercetin (m/z 303.1-- > 153.1). The electrospray voltage was set to 5500 V; the heater was set at 600°C; the curtain gas was at 35 psi; and GS1 and GS2 were both at 60 psi. Analysis of each sample was repeated three times using three biological replicates.

## Abbreviations

AN2: Anthocyandin; ANS: Anthocyanidin synthase; 2bHLH: basic helix-loop-helix; CHI: Chalcone flavanone isomerase; CHS: Chalcone synthase; 4CL: 4-Coumarate:coa ligase; C4H: Cinnamate 4-hydroxylase; COMT: Catechol-o-methyltransferase; DAP: Days after pollination; DFR: Dihydroflavonol reductase; MYB: Myeloblast; PAP-1: Production of anthocyanin pigment1; SSR: Simple sequence repeat; TF: Transcription factor; UFGT: *Udp glucose-flavonoid 3-o-glucosyl transferase.*

## Competing interests

The authors declare that they have no competing interests.

## Authors’ contributions

SV: Conceptualization of experiments, sequence characterization, mapping, functional analysis of *MdMYB3,* flavonoid analysis*,* writing of manuscript*;* YH: conceptualization of experiments, mapping, and critical revision of manuscript; GW: expression analysis experiments with multiple genotypes of apple; SSK: conceptualization of experiments; critical revision of manuscript. All authors read and approved the final manuscript.

## Supplementary Material

Additional file 1: Table S1 List of primer sequences for real-time PCR. Click here for file
