# Supplementary material for: An apple MYB transcription factor, MdMYB3, is involved in regulation of anthocyanin biosynthesis and flower development
Source: BMC Plant Biol. 2013 Nov 7;13:176. doi: 10.1186/1471-2229-13-176 (PMC3833268; doi:10.1186/1471-2229-13-176)
Supplement: Additional file 1: Table S1 — List of primer sequences for real-time PCR. [file 1471-2229-13-176-S1.docx]

**Table S1. List of primer sequences for real-time PCR.**

| **Genus** | **Gene name** | **Gene identifier** | **Forward primer** | **Reverse primer** |
| --- | --- | --- | --- | --- |
| *Malus* | *MdMYB4* |  | GGCAAGAGTTGCAGGTTGAG | GTCGCTTGATGTGTGTGTTCC |
|  | *MdCHS* | X68977 | TCAAGCCTATTGGGATTTCG | CAGCTGACTTCCTCCTCACC |
|  | *MdCHI* | X68978 | GATATCGAAGCCGGAAATGA | TGTTGACTCACGCCAACAAT |
|  | *MdF3H* | AF117270 | ACACCAAATATGGCTCCTGC | TTTCGTTGCTGAAGTCGTTG |
|  | *MdFLS* | AF119095 | AATGGGAGTGGAGTCTGTGG | AGTTGGAGCTGGCCTCAGTA |
|  | *MdDFR* | AF117268 | AAGGCCGTTACATTTGTTCG | GCCCTTGAACTTTGTGGGTA |
|  | *MdUFGT* | AF117267 | AGCTCCACTCGGAACTTCAA | AACCCGCCCTAAATATGTCC |
|  | *MdANS* | AF117269 | CAATTTGGCCTCAAACACCT | TCAACACCAAGTGCAAGCTC |
|  | *MdANR* | DQ099803 | GTTGCAACCCCTGTCAACTT | CACGACCAAACCTGTTCCTT |
|  | *MdLAR1* | DQ139836 | ACAACACCCACCCTTCTGAG | TGCAGCAAGGGCTAGTAGGT |
|  | *MdActin* | DQ822466 | CTACAAAGTCATCGTCCAGACAT | TGGGATGACATGGAGAAGATT |
| *Nicotiana* | *NbActin* | AY179605 | AATGATCGGAATGGAAGCTG | TGGTACCACCACTGAGGACA |
|  | *NtCHI* | AB213651 | GAAATCCTCCGATCCAGTGA | CAACGTTGACAACATCAGGC |
|  | *NbCHS* | EF421432 | AGAAAAGCCTTGTGGAAGCA | ACTTGGTCCAAAATTGCAGG |
|  | *NtF3H* | AB289450 | ACAGGGTGAAGTGGTCCAAG | CCTTGGTTAAGGCCTCCTTC |
|  | *NtF3'H* | AB289449 | TCCAAGAATACTGGCCCAAG | CTCACAACTCTCGGATGCAA |
|  | *NtFLS* | AB289451 | GAACTTGAAGGGAAAAGGGG | TCCCTGTAGGAGGGAGGATT |
|  | *NbDFR1* | EF421431 | TCCCATCATGCGATCATCTA | ATGGCTTCTTTGTCACGTCC |
|  | *NtLAR* | AM827419 | TCAAGGTCCTTTACGCCATC | ACGAACCTGCTTCTCTTTGG |
|  | *NtANS* | AB289447 | TGGCGTTGAAGCTCATACTG | TTTCAAGGGTGTCCCCAATA |
|  | *NtUFGT* | FG627024 | GAGTGCATTGGATGCCTTTT | CCAGCTCCATTAGGTCCTTG |
|  | *NtANR1* | AM791704 | CATTTGACTTTCCCAAACGC | ATTGGGCTTTTGAGTTGTGC |
|  | *NtANR2* | DW003895 | TGTTCCCACTTGGGATGATA | TGCACCTATACTCTGTTAGTGGC |
|  | *NtC4H* | AB236952 | CCAGGAGTGCAAGTGACTGA | ACCACCAAGCGTTAACCAAG |
|  | *NtPAL* | AB289452 | CCTCAGAACATCACCCCAGT | ACCGTGTAACGCCTTGTTTC |
|  | *NtCOMT* | Z56282 | TTTTCGTGGATGCTGACAAG | GGGTAATTCCATCACCAACG |
|  | *NtCAD* | AY911854 | CGAAGACATTGGCTGAGGAT | TTGGGTATGTTTCAGCACCA |
